# Writing of H3K4Me3 overcomes epigenetic silencing in a sustained but context-dependent manner

**DOI:** 10.1038/ncomms12284

**Published:** 2016-08-10

**Authors:** David Cano-Rodriguez, Rutger A F. Gjaltema, Laura J Jilderda, Pytrick Jellema, Jelleke Dokter-Fokkens, Marcel H J. Ruiters, Marianne G Rots

**Affiliations:** 1 Epigenetic Editing Research Group, Department of Pathology and Medical Biology, University of Groningen, University Medical Centre Groningen, Hanzeplein 1, 9713 GZ, Groningen, The Netherlands

## Abstract

Histone modifications reflect gene activity, but the relationship between cause and consequence of transcriptional control is heavily debated. Recent developments in rewriting local histone codes of endogenous genes elucidated instructiveness of certain marks in regulating gene expression. Maintenance of such repressive epigenome editing is controversial, while stable reactivation is still largely unexplored. Here we demonstrate sustained gene re-expression using two types of engineered DNA-binding domains fused to a H3K4 methyltransferase. Local induction of H3K4me3 is sufficient to allow re-expression of silenced target genes in various cell types. Maintenance of the re-expression is achieved, but strongly depends on the chromatin microenvironment (that is, DNA methylation status). We further identify H3K79me to be essential in allowing stable gene re-expression, confirming its role in epigenetic crosstalk for stable reactivation. Our approach uncovers potent epigenetic modifications to be directly written onto genomic loci to stably activate any given gene.

## Abstract (web summary)

Epigenome editing by zinc finger (ZF) and CRISPR-dCas9 technologies can induce or repress gene expression. Here, the authors show that histone methyltransferase PRDM9 fused to either dCas9 or ZF proteins can sustain gene re-expression, and H3K79me is required for stable gene re-expression.

## Graphical abstract

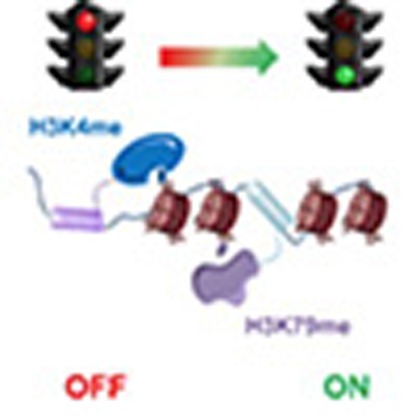

Epigenetics controls gene expression patterns in a cell-specific and mitotically stable manner. Genome-wide analysis and gene expression profiling studies identified specific combinations of modifications of DNA and histones, as well as transcriptional regulators, to correlate with chromatin accessibility and expression^1^,^2^,^3^. For example, methylation of lysine residue 4 or 79 on histone H3 (H3K4me3 and H3K79me2–3) or monoubiquitination of histone H2B (H2Bub1) situated at transcription start sites (TSSs) are associated with transcriptionally active euchromatin^4^,^5^,^6^,^7^,^8^,^9^,^10^. On the other hand, DNA methylation in core promoter regions is mainly involved in gene silencing. Elucidating the distinction between the mere associative presence versus the actual causality of transcription by chromatin marks is an important area of investigation^11^,^12^. Current approaches to studying chromatin function often make use of small-molecule inhibitors and RNA interference to unravel the role of epigenetic enzymes in transcription regulation. Although these studies have yielded basic insights into epigenetic regulation, they are hampered by genome-wide effects^13^,^14^. Identifying the conditions that drive transcriptional changes is critical to understanding how cell identity is established and how genes become permanently dysregulated in human diseases.

An innovative approach to study transcriptional changes is by synthetic modulation of gene expression. Gene expression modulation can be achieved using artificial transcription factors by coupling transcriptional activators or repressors to DNA-targeting platforms such as zinc-finger (ZF) domains, transcription activator-like effector domains and the clustered regularly interspaced palindromic repeats (CRISPR–dCas)^15^,^16^,^17^,^18^,^19^,^20^,^21^,^22^,^23^,^24^,^25^,^26^,^27^,^28^,^29^. Even though changes in gene expression have been successful, the sustainability of such induced transcriptional reprogramming is still under debate. Indeed, these artificial systems merely act as scaffolds to recruit multiple transcriptional components and have no enzymatic activity on the chromatin state directly. Therefore, methods for directly linking transcriptional function with the presence or absence of epigenetic marks are needed to establish general principles for (sustained) cell reprogramming. One elegant method to establish those general rules is epigenome editing^30^,^31^,^32^,^33^. Since the dynamic remodelling of the chromatin landscape is tightly regulated by a conglomerate of enzymes and macromolecules, there is an extensive array of epigenetic effector domains suitable for gene expression modulation^34^,^35^,^36^.

Several studies have already shown the potency of epigenome editing in inducing^37^,^38^,^39^,^40^,^41^,^42^ or repressing^41^,^42^,^43^,^44^,^45^,^46^,^47^,^48^,^49^ gene expression. Despite the fact that gene expression could be modulated, little is known about the stability of the acquired epigenetic states. In this respect, gene repression by DNA methylation has been shown to be stable and heritable using engineered ZFs fused to DNA methyltransferases to target the *SOX2* gene^46^, while the repressive effect was not achieved in another context for the *VEGF-A* gene^49^. In contrast, sustained gene reactivation remains largely unexplored. Studies so far have been focusing on activating gene expression by VP64-based artificial transcription factors and, in a few cases, epigenetic enzymes (TET and p300)^37^,^38^,^39^. Although gene induction has been achieved, the sustainability of this overexpression has not been documented yet. To fully exploit the potentials of epigenome editing, it is necessary to understand how the chromatin microenvironment affects mitotic stability of reprogrammed gene expression patterns.

Trimethylation of H3K4 is a hallmark of gene expression and the presence of this mark at promoters of protein coding genes might serve as a transcriptional on-off switch^50^. H3K4me3 is found in ∼75% of all human active gene promoters in several cell types, suggesting that it plays a key role in mammalian gene expression^2^. Here we have employed epigenome editing to investigate the role and stability of H3K4me3 in transcriptional activation. In light of the fact that H3K4me3 at TSSs is frequently associated with active transcription, we aimed to achieve targeted gene re-expression of epigenetically silenced genes by local induction of this mark. Using the histone methyltransferase PRDM9 (refs 51, 52, 53) fused to either dCas9 or ZF proteins, we examined the role of H3K4me3 in upregulating the expression of several model genes in different chromatin contexts. We also identified potential reinforcing marks to achieve stable gene activation. As such, our study identified H3K4me3 and H3K79me as well as the absence of DNA methylation to be critical in allowing sustained re-expression of epigenetically silenced genes.

## Results

### Effect on gene expression by PRDM9 induced H3K4me3

**Figure 1:**
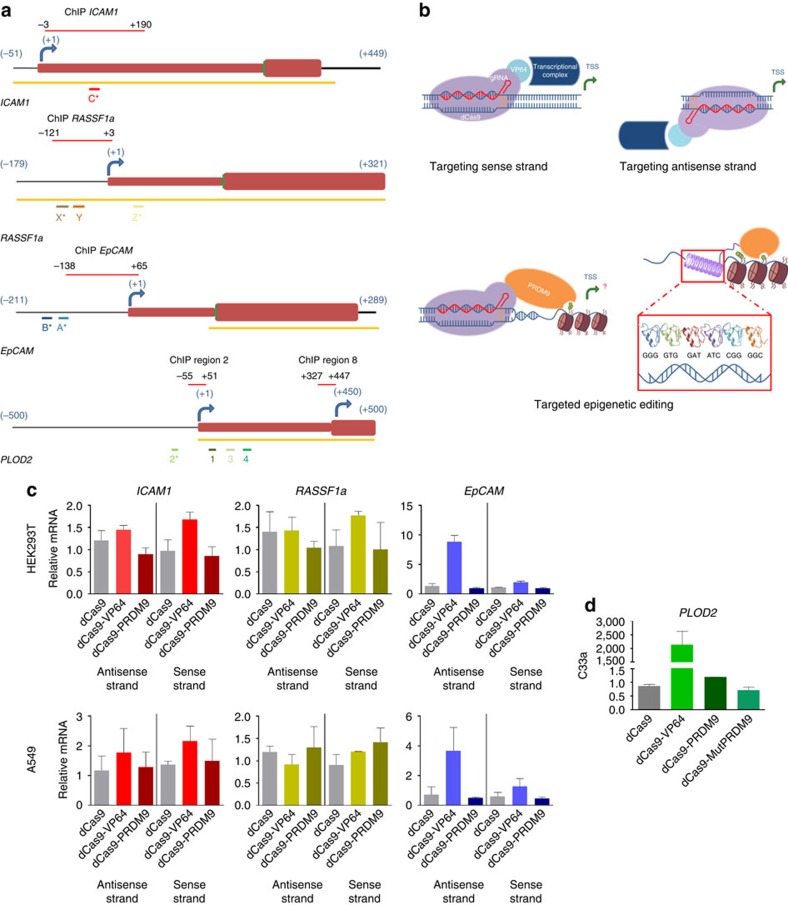
Local induction of H3K4me3 activates transcription of endogenous genes from promoter regions using CRISPR–dCas9. (**a**) Schematic representation of the targeted genes and the (overlapping) locations where the ZFs and gRNAs bind (the letter or number of each region refers to the name of the ZF or gRNA, for the regions marked with * a ZF as well as a gRNA were designed). The yellow bars represent the location of the CpG islands. (**b**) Schematic of dCas9-VP64 targeting sense and antisense strands of DNA, and dCas9 and ZF fused to the epigenetic editor PRDM9 to locally induce H3K4me3. (**c**) Relative messenger RNA (mRNA) expression of *ICAM1*, *RASSF1a* and *EpCAM* in HEK293T and A549 cells, and (**d**) *PLOD2* in C33a cells, by the indicated dCas9 fusion protein co-transfected with a combination gRNAs targeted to each promoter region. (*n*=3 independent experiments; error bars±s.d.).

To investigate the potency of H3K4me3 in inducing gene expression, we fused the SET domain of the human PRDM9 to dCas9. We transiently co-transfected HEK293T and A549 cells to express the proteins (dCas9-empty, the transcriptional activator dCas9-VP64 and dCas9-PRDM9) with a combination of guide RNAs (gRNAs) to activate the endogenous promoters of intercellular adhesion molecule 1 (*ICAM1*), Ras association domain-containing protein 1 (*RASSF1a*) or epithelial cell adhesion molecule (*EpCAM*; Fig. 1a,b). We used a combination of gRNAs to target each promoter based on previous reports indicating that multiple gRNAs at a single promoter are more effective for gene activation^15^,^16^,^18^,^19^,^21^,^22^. dCas9-VP64 was able to induce *EpCAM* gene expression in both cell lines, whereas gRNA-directed dCas-PRDM9 and dCas-VP64 were ineffective in activating *ICAM1* and *RASSF1a* (Fig. 1c). There were no clear beneficial effects when changing the binding orientation of the gRNAs. Analysis of ENCODE data depicted that the target regions of *ICAM1* and *RASSF1a* in both HEK293T and A549 were hypermethylated, not associated with H3K4me3 marks and lacked DNAse hypersensitive sites, whereas the promoters of *EpCAM* were unmethylated and contained H3K4me3 peaks (Supplementary Fig. 1a). We confirmed these differential DNA methylation levels of our three model genes around the promoter area in both cell lines (Supplementary Fig. 1b). This suggested that the dCas9 is not able to access the promoters of hypermethylated genes, explaining the lack of effect of VP64 and the PRDM9 catalytic domain. To further confirm this, we choose to target procollagen-lysine, 2-oxoglutarate 5-dioxygenase 2 (*PLOD2*) in C33a cells, which is transcriptionally repressed, although its promoter has low DNA methylation levels (Supplementary Fig. 1b). Indeed, dCas9-VP64 was able to induce high levels of gene transcription from the endogenous *PLOD2* promoter (≈2,000-fold) (Fig. 1d). In addition, dCas9-PRDM9 was able to moderately but significantly upregulate *PLOD2* expression up to 1.7-fold compared with its catalytically inactive mutant (MutPRDM9; *P*<0.05, two-tailed unpaired *t*-test), which did not change the target gene expression. To test whether the actual binding of dCas9 was indeed impaired by DNA hypermethylation, we performed anti-FLAG chromatin immunoprecipitation (ChIP) using cells transfected with dCas9-3XFLAG and a combination of gRNAs for the different cell lines and genes (Supplementary Fig. 1c). dCas9 is not able to efficiently bind regions located in CpG islands (CGIs), where DNA hypermethylation is present (*ICAM1* and *RASSF1a*) as compared with regions outside of CGIs or without DNA hypermethylation (*EpCAM* and *PLOD2*). Taken together, our data suggests that DNA hypermethylation of CGIs severely hampers the binding or effect of dCas9 fusions. Importantly, for a susceptible silenced locus, H3K4me3 could directly induce gene expression.

**Figure 2:**
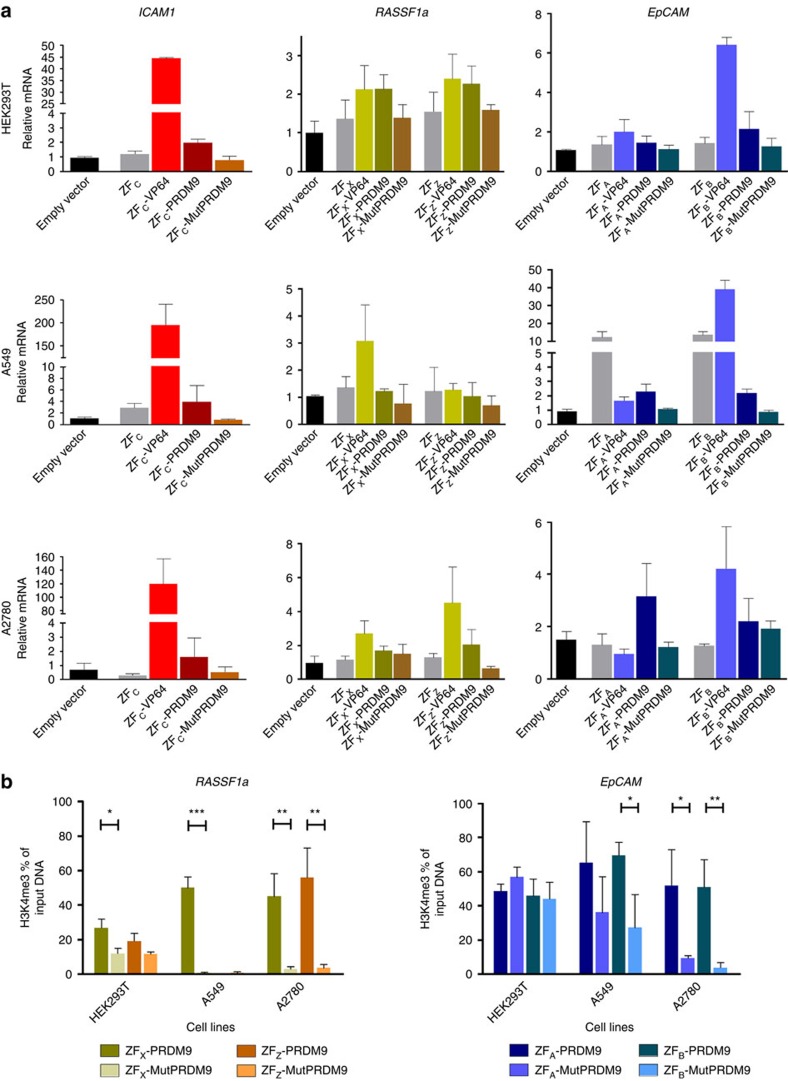
Local induction of H3K4me3 activates transcription of endogenous genes from promoter regions using zinc-finger proteins. (**a**) Relative messenger RNA (mRNA) expression of *ICAM1*, *RASSF1a* and *EpCAM* in HEK293T, A549 and A2780 cells determined by qRT–PCR, induced by the indicated ZF fusion protein after retroviral transduction, the letter of each ZF corresponds to the same region where a gRNA binds at the promoter. (**b**) H3K4me3 ChIP–qPCR enrichment at the promoter region of *EpCAM* and *RASSF1a* in HEK293T, A549 and A2780 after retroviral transduction with ZF-PRDM9 and ZF-MutPRDM9 (two-tailed unpaired *t*-test, *P<0.05, **P<0.01, ***P<0.001). *n*=3 independent experiments; error bars±s.d.

To further confirm the effects of PRDM9, we analysed the targeting of *ICAM1, RASSF1a* and *EpCAM* using the smaller ZF proteins targeting the same regions as the gRNAs. In the two cell lines (HEK293T and A549) and an additional cell line with hypermethylation in the three genes (A2780; Supplementary Fig. 1d), individual ZF-VP64 fusions were able to upregulate *EpCAM*, *ICAM1* and *RASSF1a* gene expression, but the effect of PRDM9 was at the most subtle, reaching significance compared with the mutant for *ICAM1* in HEK293 and both ZF_A_ and ZF_B_ (*EpCAM)* in A549 (Fig. 2a). We thus assessed the capability of the ZF-PRDM9 fusions to induce H3K4me3 editing in these cells (Fig. 2b); PRDM9 was able to efficiently increase H3K4 trimethylation on *EpCAM* and *RASSF1a* promoters (up to 60%) compared with the mutant, despite the DNA hypermethylation. This editing could not be achieved when H3K4me3 was already enriched at the TSS (that is, for *EpCAM* in HEK293T).

### Sustained reactivation by H3K4me3 is dependent on DNA methylation

**Figure 3:**
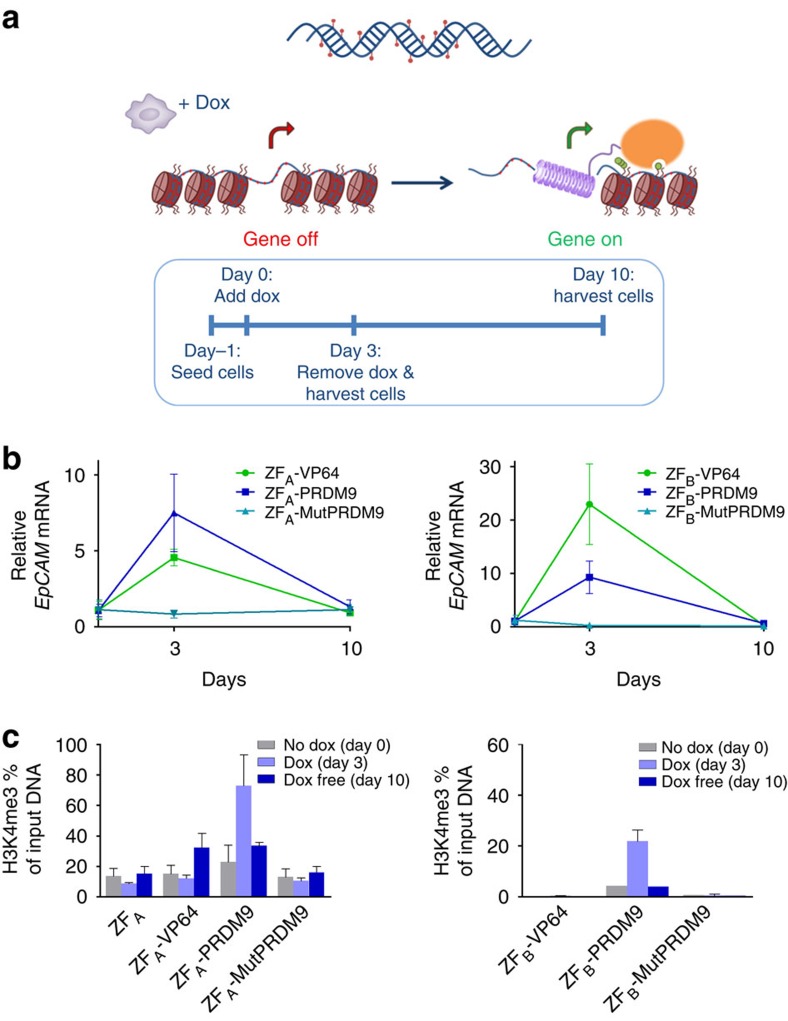
Stability and maintenance of gene reactivation after induction of H3K4me3 in the hypermethylated *EPCAM* gene in HeLa cells. (**a**) Schematic representation of the stable doxycycline inducible system and the experimental timeline set-up. (**b**) Relative *EpCAM* messenger RNA (mRNA) expression, at each specific time point using two different ZFs targeting the promoter region. (**c**) H3K4me3 ChIP–qPCR enrichment at the promoter region of *EpCAM* at each specific time point. *n*=3 independent experiments; error bars±s.d.

To fully address effectivity and sustainability of gene activation, we engineered a stable inducible system to target the hypermethylated *EpCAM* gene in HeLa cells (Fig. 3a). Again two different regions of the *EpCAM* promoter were targeted (ZF_A_ and ZF_B_) outside of the CGI. Doxycycline (Dox) treatment to express ZF_A_ fusions for 3 days resulted in *EpCAM* re-expression using both the transcriptional activator VP64 (≈5-fold) and PRDM9 (≈8-fold; Fig. 3b). However, *EpCAM* expression decayed to background levels after Dox removal and subculturing for 7 additional days. For ZF_B_, similar patterns were obtained. To confirm active epigenome editing, we determined the presence of H3K4me3 using ChIP–quantitative PCR (qPCR; Fig. 3c). Background levels of ∼18% were present as seen for ZF_A_ only, mutPRDM9 and the no Dox controls. High levels of H3K4me3 (up to 75%) were achieved when the ZF_A_-PRDM9 was expressed for 3 days (*P*<0.05 compared with no Dox, two-tailed unpaired *t*-test). In contrast, 3-day expression of ZF_A_-VP64 was not able to induce the active histone mark. So, although active transcription is thought to drive trimethylation of H3K4, we instead show that local writing of this mark is able to directly initiate gene expression. Remarkably, we were not able to achieve sustained gene re-expression under this chromatin context for both treatments after 7 days of subculturing (day 10).

**Figure 4:**
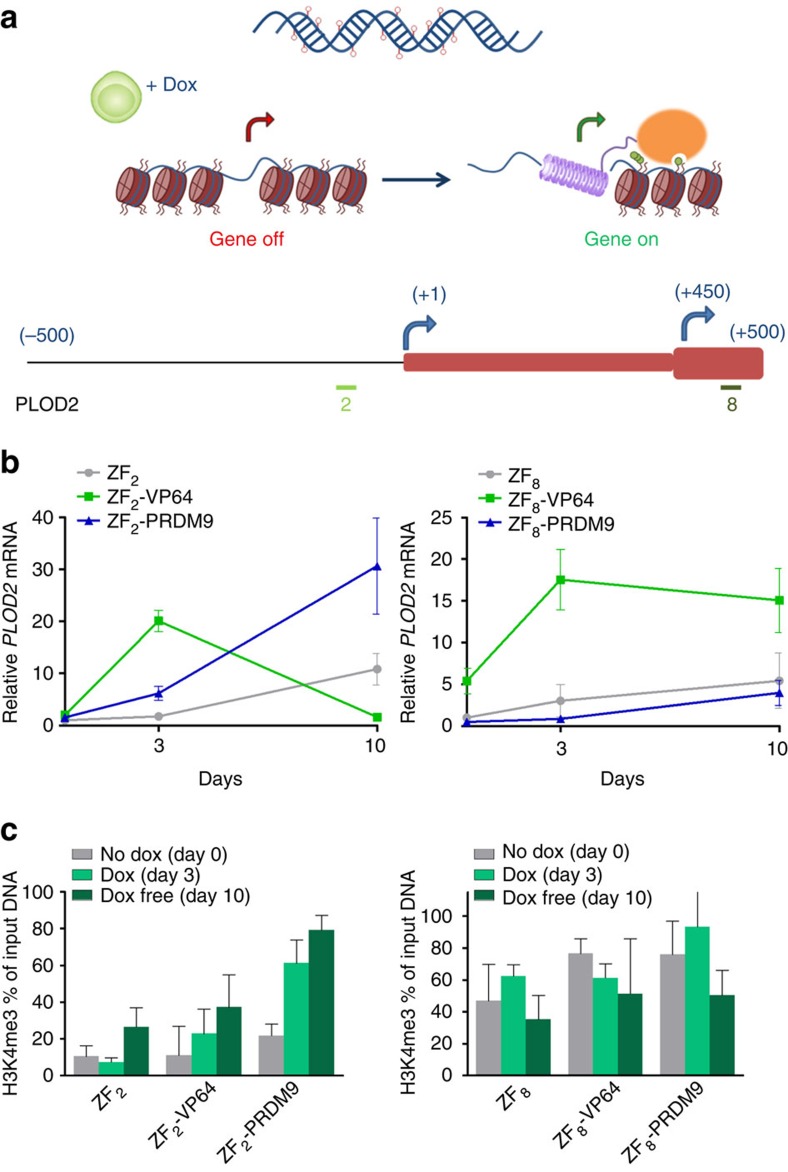
Stability and maintenance of gene reactivation after induction of H3K4me3 in the repressed non-methylated *PLOD2* gene in C33a cells. (**a**) Schematic representation of the stable doxycycline inducible system and the two regions targeted by the ZF protein fusions. (**b**) Relative *PLOD2* messenger RNA (mRNA) expression, at each specific time point using two different ZFs targeting the promoter region. (**c**) H3K4me3 ChIP–qPCR enrichment at the promoter region of *PLOD2* at each specific time point. *n*=3 independent experiments; error bars±s.d.

Since previous data suggest that H3K4me3 and DNA methylation are mutually exclusive^54^, we hypothesized that the presence of DNA methylation in highly dense CGIs interfered with sustained gene re-expression induced by PRDM9. To examine this, we used the repressed but non-hypermethylated *PLOD2* gene in C33a cells as our model, and addressed the sustainability of gene expression. Using the Dox-inducible system, we targeted two TSSs in the *PLOD2* gene (Fig. 4a). When targeting the region of the first TSS (ZF_2_), we were able to induce *PLOD2* expression using VP64 and PRDM9 upon 3 days exposure to Dox (≈20- and ≈6-fold, respectively; Fig. 4b). Remarkably, gene reactivation was sustained and even reinforced (≈30-fold) after Dox removal and subculturing the cells for 7 days, when using PRDM9. For VP64, gene expression levels returned to background after subculturing. Targeting the second TSS (ZF_8_) using PRDM9 had no effect on gene expression, although ZF_8_-VP64 induced expression that was maintained for 7 days. We analysed the efficacy of H3K4me3 editing after targeting the two locations over time (Fig. 4c): trimethylation of H3K4 was locally induced when targeting PRDM9 to region 2 (60–80%, *P*<0.01 compared with no Dox, two-tailed unpaired *t*-test), but no further increase was achieved for region 8, where background levels were as high as 35–40%. After subculturing, the H3K4me3 was sustained and reinforced for the ZF targeting region 2 (reflecting gene expression levels), while no clear difference was observed for region 8. The H3K4me3 enrichment was evaluated at a region 750 bp upstream of TSS to test for the spreading of the mark (Supplementary Fig. 3a). H3K4me3 seems to spread after subculturing the cells without dox.

### H3K79me is involved in sustained gene re-expression

**Figure 5:**
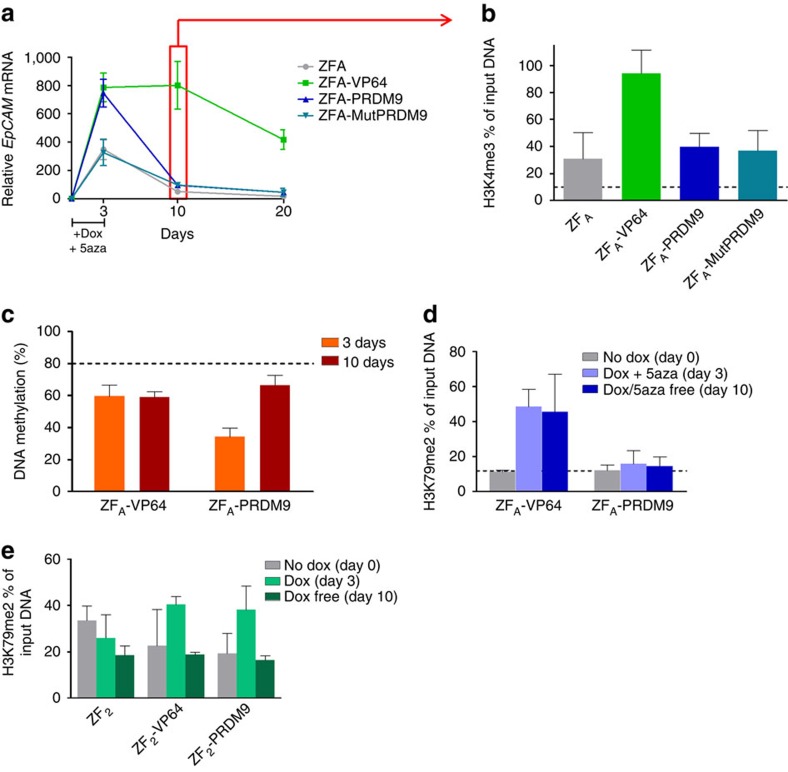
Stability and maintenance of H3K4me3 after DNA demethylation. (**a**) Relative *EpCAM* messenger RNA (mRNA) expression, at each specific time point after using the inhibitor of DNA demethyltransferases 5′aza for 3 days. (**b**) H3K4me3 ChIP–qPCR enrichment at the promoter region of *EpCAM* 10 days after demethylation and ZF fusion protein expression. (**c**) DNA methylation levels at the *EpCAM* promoter determined by pyrosequencing, black-dot line represents mean methylation levels of untreated cells. (**d**) H3K79me2 ChIP–qPCR enrichment at the promoter region of *EpCAM* 3 and 10 days after demethylation and ZF fusion protein expression. (**e**) H3K79me2 ChIP–qPCR enrichment at the promoter region of *PLOD2* 3 and 10 days after ZF fusion protein expression. *n*=3 independent experiments; error bars±s.d.

H3K4me3 can prevent DNA methyltransferase binding^54^. To test whether DNA methylation also has inhibitory effects on the sustainability of locally enforced H3K4me3, we treated our inducible *EpCAM* HeLa cell lines with an inhibitor of DNA methyltranferase activity 5-Aza-2′-deoxycytidine (5′aza). The induced DNA demethylation resulted in higher levels of expression after 3 days of expression for all fusions, also for ZF_A_ only and ZF_A_-MutPRDM9 (Fig. 5a). Surprisingly, only ZF_A_-VP64 was able to achieve sustained *EpCAM* re-expression, which lasted at least 20 days, while cells targeted with the ZF_A_ alone, PRDM9 or the inactive mutant returned to the repressed state. The same effect was observed when depleting *UHRF1*, involved in the maintenance of DNA methylation after DNA synthesis (Supplementary Fig. 2a,b).

To address whether the presence of H3K4me3 was sustained after DNA demethylation, we performed ChIP–qPCR at day 10 after Dox and 5′aza treatment. After DNA demethylation, cells expressing ZF_A_-VP64 showed high levels of H3K4me3, in comparison with the other cells (Fig. 5b). DNA demethylation was not completely effective as determined by pyrosequencing (20–40% less than untreated cells, which demonstrated DNA methylation levels of ∼80%). Interestingly, the levels of methylation increased after 10 days for PRDM9, but were maintained low for VP64 (Fig. 5c). It has been previously shown that the lack of H3K79me resulted in incomplete reactivation of tumour suppressor genes after treating with inhibitors of DNA methyltransferases^55^. For this reason, we checked the presence of H3K79me2 in the cells treated with 5′aza. The presence of H3K79me2 at day 3 was increased upon targeting of the transcriptional activator VP64, but not for the PRDM9-expressing cells (Fig. 5d). This supports the indication that H3K79me is required for the stability and maintenance of H3K4me3. We determined the presence of H3K79me2 in the stable C33a cells targeting *PLOD2* around the first TSS, where targeting of PRDM9 achieved sustained re-expression. We indeed observed the presence of the mark (Fig. 5e), which urged us to address the role of H3K79me in allowing stability of H3K4me.

### Co-editing of H3K4me and H3K79me to maintain re-expression

**Figure 6:**
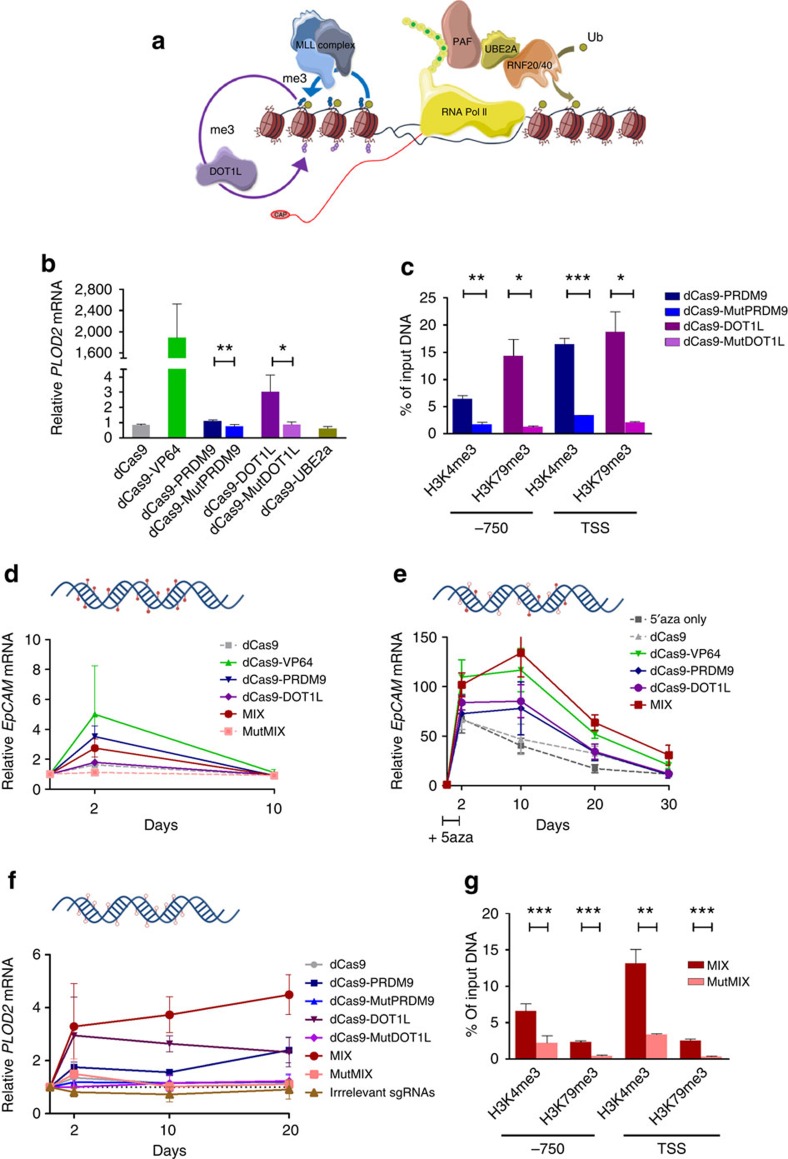
Achieving sustained gene re-expression using different epigenetic editors. (**a**) Graphical representation of the process of gene transcription with the main epigenetic players; RNA polymerase II recruits the ubiquitin-conjugating and -ligating enzyme via PAF to monoubiquitinate H2B, this ubiquitination is required for H3K4me3 and H3K79me. (**b**) Relative *PLOD2* messenger RNA (mRNA) expression, after co-transfection of dCas9 fusions and a combination of gRNAs. (**c**) H3K4me3 and H3K79me3 ChIP–qPCR enrichment at the promoter region of *PLOD2* (around TSS and 750 bp upstream. (**d**) Relative *EpCAM* mRNA, after co-transfection in HeLa cells of dCas9 fusions and a combination of gRNAs. (**e**) Relative EpCAM mRNA, after co-transfection in HeLa cells of dCas9 fusions and a combination of gRNAs and 5′aza treatment. (**f**) Relative *PLOD2* mRNA, after co-transfection in C33a cells of dCas9 fusions and a combination of gRNAs. (**g**) H3K4me3 and H3K79me3 ChIP–qPCR enrichment at the promoter region of *PLOD2* 20 days after seeding cells around TSS and 750 bp upstream (two-tailed unpaired *t*-test, *P<0.05, **P<0.01, ***P<0.001). *n*=3 independent experiments; error bars±s.d.

To address our hypothesis that H3K79Me facilitates maintenance of H3K4Me3-induced gene expression, we took advantage of the CRISPR–dCas9 system to co-recruit various key transcriptional activating epigenetic candidates (Fig. 6a). The mechanism of H3K4me3 deposition has been well documented and requires *cis* and *trans* histone post-translational modification crosstalk. For example, H2B monoubiquitination is required for H3K4me3 and H3K79me formation^9^,^10^. This process requires the E2 ubiquitin-conjugating enzyme (UBE2A) in association with the E3 ubiquitin-ligating enzymes RNF20 or RNF40. Monoubiquitination is then recognized by the transcriptional machinery and recruits H3K4 methyltransferases as well as DOT1L (H3K79 methyltransferase).

To confirm gene expression modulation via epigenetic editing using the dCas9 platform, we first targeted the *PLOD2* promoter in C33a cells with the three different enzymes: PRMD9 (or its mutant), UBE2A or DOT1L (or its mutant). Upon co-transfection of a dCas fusion plasmid with a combination of gRNA plasmids, dCas9-PRDM9 and dCas9-DOT1L were capable of achieving *PLOD2* gene re-expression, in contrast to mutPRDM9 or mutDOT1L (*P*<0.01 and *P*<0.05, respectively, two-tailed unpaired *t*-test). Both mutants resulted in similar gene expression as obtained upon targeting of dCas9 alone or in fusion with UBE2A (Fig. 6b). PRDM9 and DOT1L effectively deposited their intended histone mark around the TSS (16% enrichment of H3K4Me3 by dCas-PRDM9 and 18% enrichment of H3K79Me3 by dCas-DOT1L; Fig. 6c). This enrichment was evaluated at two different regions (close to TSS and 750 bp upstream). While H3K4me3 preferentially associated close to the TSS, H3K79me3 was also enriched at the upstream region.

Next, we validated our observation using ZF targeting for the hypermethylated gene *EpCAM* (Fig. 2) with a combination of gRNAs and the dCas fusions. We confirmed the expression upregulation at day 2, and sudden drop to background levels after 10 days (Fig. 6d). Similar to the ZF experiments, treatment with 5′aza resulted in prolonged re-expression until day 10, after which the expression level declined similar to 5′-aza-only expression levels for dCas-PRDM9 fusion construct (Fig. 6e). Targeting of DOT1L to *EpCAM* induced expression levels with similar kinetics as targeting PRDM1. Interestingly, when we used 5′aza in combination with a mix of dCas9-PRDM9 and dCas9-DOT1L (MIX), an effective *EpCAM* re-expression was obtained and the onset of repression of was delayed.

To further address the role of DNA methylation and the re-enforcing effects by H3K79Me, we targeted the *PLOD2* promoter in C33a cells with our panel of effectors. The dCas9-PRDM9 or dCas9-DOT1L fusion was capable of achieving *PLOD2* gene re-expression, in contrast to their respective mutants (Fig. 6f). Despite halving the amount of plasmid per construct in the MIX experiments, the combination of dCas9-PRDM9 and dCas9-DOT1L effectively induced *PLOD2* gene expression and the effect was even further improved 20 days after transfection (*P*<0.01, compared with MutMIX control, two-tailed unpaired *t*-test). A control containing a mix of irrelevant gRNAs did not show any effect on gene expression when targeting a mix of PRDM9 and DOT1L. dCas9-VP64-expressing cells overexpressed *PLOD2* after 2 days, but the effect was transient (Supplementary Fig. 4c), as obtained before for the ZF-VP64. Finally, we demonstrated that the epigenetic reprogramming was still present 20 days after induction in cells treated to express the combination of PRDM9 and DOT1L, and not in cells transfected to express the mutant combination (Fig. 6g).

## Discussion

Recent advances in engineered DNA-binding domains have opened new avenues to screen various epigenetic writers or erasers and to study their effect in transcription regulation. Even though several genes have been targeted for reactivation using epigenetic editors, inducing gene expression in a mitotically stable manner has not been addressed^36^,^37^,^38^,^39^,^40^,^41^. Given the tight association of H3K4me3 and promoter activity, we set out to induce permanent gene re-expression by local enrichment of this mark. We clearly established a causative role of H3K4me3 in instructing gene transcription, a notable finding of our study. Moreover, our results suggest that gene re-expression achieved by epigenetic editing can be maintained in DNA hypomethylated loci, in contrast to hypermethylated loci. Thus, we neatly indicate that the chromatin microenvironment affects the long-term effects of epigenome editing.

Targeted trimethylation of H3K4 was sufficient to activate endogenous gene expression, indicating that this mark is instructive in the transcriptional process. This finding fuels the debate of cause-consequence of gene expression and supports recent studies that show the role of H3K4me3 to facilitate the transcriptional pre-initiation complex formation by recruiting the transcription factor machinery via the TAF3 subunit of TFIIH (refs 56, 57). Fusion of the catalytic core domain of PRDM9 to dCas9 and ZFs resulted in transactivation of target genes when compared with the DNA-binding domain alone or the catalytically inactive mutant. Importantly, only PRDM9, and not VP64, was capable of both transcriptional activation and enrichment of the mark at promoters targeted. Using the catalytic domain of PRDM9, a protein involved only in H3K4me3 events during meiosis, we minimized the chances of inducing the mark by recruiting other factors. The effect of PRDM9 was more subtle than the transcriptional activator VP64, which may be due to the differences in size, as the size effects are also observed when comparing ZFs and dCas9. Alternatively, H3K4me3 by itself might have a low capacity to actively induce gene expression when is not accompanied by other factors. VP64, for example, is able to recruit co-factors, including p300 (ref. 37) and chromatin remodelers, which in turn allows for higher levels of transcription.

The indication that DNA hypermethylation in promoters with highly dense CGIs is impairing the capacity of transactivation by dCas9 is an important finding in our study and warrants systematic research. Epigenetic features such as CpG methylation and chromatin accessibility are reported to have little effect on targeting^21^,^22^,^58^. However, although these studies indeed achieved gene upregulation using dCas9 for repressed genes, no target loci within CGIs were included. Here we provide indications that the targeting of regions embedded in hypermethylated CGIs strongly hampers binding of dCas9. Indeed, targeting the hypermethylated *EpCAM* gene in HeLa cells by dCas fusions could successfully induce gene expression, as the gRNAs were designed to bind outside of the dense CGI. Our data are in line with the findings from genome-wide screenings, demonstrating that next to DNAse hypersensitivity, the frequency of CG or GC dinucleotides reflect aspects involved in dCas9 binding^59^. Also, a more recent study proved that Cas9 binding is impaired by nucleosome density *in vivo* and *in vitro*^60^. Indeed, CGI promoters when methylated are often nucleosome occupied^61^. Altogether, methylation status should be taken into account when targeting CpG-rich hypermethylated regions using the dCas platform. In this respect, ZF proteins seem to have the capacity to fully bind and exert an effect in the local chromatin regardless of the chromatin microenvironment, as also extensively reported for by us^23^,^26^,^28^,^38^ and others^25^,^29^,^40^.

**Figure 7:**
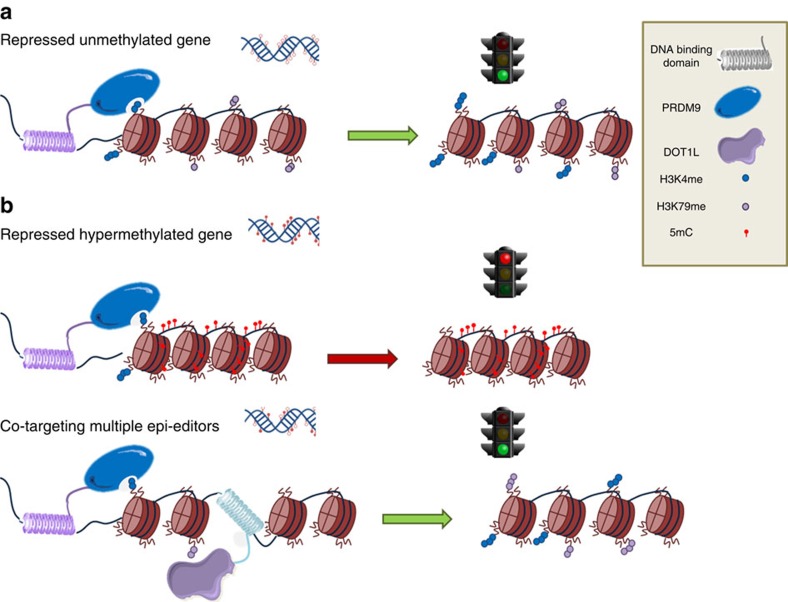
Model of epigenome editing to achieve stable gene reactivation depending on the chromatin microenvironment (**a**) Stable gene reactivation is achieved by targeting H3K4 methyltransferases to a non-hypermethylated locus. (**b**) Gene reactivation is not achieved by targeting H3K4 methyltransferases to a hypermethylated locus. (**c**) Co-targeting different epigenetic editors to achieve sustained reactivation at hypermethylated locus.

The technology of epigenome editing allows addressing the dynamics of histone crosstalk and sustainability of endogenous gene re-expression. Using a combination of effector domains and epigenetic drugs, we were able to unravel key events necessary to induce stable gene re-expression. We further confirmed post-translational modification crosstalk between histones, demonstrating synergy by the complementary effect of H3K4me3 and H3K79me. The model that we propose underlines the importance of knowing the local chromatin microenvironments at the targeted loci based on these outcomes (Fig. 7): if there is no/low DNA methylation present, targeting H3K4 methyltransferases is enough to achieve stable gene re-expression when H3K79me is present at the promoter. In contrast, when DNA methylation is present at the promoter area, writing of H3K4me3 does results in re-expression but transiently, while co-targeting of multiple effector domains is able to achieve stable gene reactivation using H3K4 and K79 methyltransferases in combination with DNA demethylation.

Understanding the dynamics of the chromatin microenvironment is important to unravel the mechanisms underlying stable gene reactivation. Here we show different outcomes for gene re-expression that are dependent on the local chromatin landscape. Targeting of H3K4me3 in a hypermethylated locus is capable of achieving only transient reactivation, while targeting a non-hypermethylated locus is enough to achieve stable reactivation. With our system, we exploited histone reinforcing crosstalks to achieve long lasting gene reactivation. Even after DNA demethylation, H3K4me3 was not maintained, which can be explained by the low levels of demethylation in our experimental system using 5′aza. The remaining methylated DNA could be sufficient to establish secondary gene repression. We also show the requirement of H3K79me for the stability of H3K4me3. This finding is in concordance with a previous study that showed the reactivation of tumour suppressor genes after 5′aza treatment allowed transient H3K4me3, but the expression was not sustained due to the absence of H3K79me (ref. 55). Histone crosstalk is an important mechanism required for gene transcription as described above. Here we demonstrate the crosstalk between H3K4me and H3K79me to play a role in the stability and maintenance of gene transcription. By targeting epigenetic effector domains to promoters, we provide for the first time functional evidence supporting the intrinsic roles of H3K4me3 and/or H3K79me marks in causing transcription.

Elucidating the mechanisms whereby histone modifications might be involved in cellular regulation is of fundamental importance in biology. However, due to the complexity of chromatin and the lack of knowledge in understanding the dynamic process of transcription, many interrogations are still unsolved. We used targeted epigenome editing to unravel the epigenetic mechanisms important for gene transcription. Our system establishes minimal epigenetic requirements to achieve long-term gene re-expression. Several technologies make use of gene expression modulation to change transcription. Manipulating gene expression at will is critical to achieve cellular reprogramming, which can be catalyst to improve different molecular biology and therapeutic applications. Sustained gene reprogramming of diseases with aberrant gene expression patterns can fulfil the promise of the curable genome. Finally, our study presents other epigenetic effector domains, to be added to the available tool set for effective epigenome editing.

## Methods

### Plasmid construction

pMLM3705 and MLM3636 plasmids were a gift from Keith Joung (Addgene plasmid #47754 and #43860, respectively). An additional multiple cloning site was added by replacing the VP64 activator in dCas9-VP64 with a sequence containing a PacI restriction site (new plasmid referred to as dCas9-Empty). The catalytic domain of human histone methyltransferase PRDM9 was amplified from total complementary DNA (cDNA) of a testicular cancer cell line, and the ubiquitin-conjugating enzyme UBE2A and histone methyltransferase DOT1L catalytic domains from human fibroblasts by Pfu DNA polymerase (Thermo Scientific, Leon-Rot, Germany), using forward and reverse primers introducing MluI and PacI restriction sites at the 5′ and 3′ ends, respectively (Supplementary Table 1; Supplementary Note 1). These catalytic domains were introduced into dCas9-Empty using sticky-end ligation after digestion with AscI and PacI digestion with T4 ligase (Thermo Scientific). Cloning of gRNAs was achieved as previously described^19^. Briefly, pairs of DNA oligonucleotides encoding 20 nt gRNA targeting sequences were annealed together to create double-stranded DNA fragments with 4 bp overhangs (Supplementary Table 2). These fragments were ligated into BsmBI-digested plasmid pMLM3636. Irrelevant gRNAs were designed to bind regions of the mouse genome. *EpCAM* targeting ZF protein (ZF_A_ and ZF_B_)^28^, *ICAM* targeting ZF protein (ZF_C_, kindly provided by C.F. Barbas III, the Scripps Institute, La Jolla, CA, USA)^25^, *RASSF1a* targeting ZF proteins (ZF_X_ and ZF_Z_; self-engineered) and *PLOD2* targeting six ZF proteins (ZF_2_ and ZF_8_)^62^ were used for epigenetic editing of the respective gene promoter (Supplementary Table 3). To replace VP64 with the catalytic domains, we used sticky-end ligation after digestion with fast-digest restriction enzymes MluI and PacI (Thermo Scientific). Each ZF effector domain construct contains a nuclear localization signal and a terminal haemagglutinin (HA) decapeptide tag. We verified all PCR-cloned constructs by DNA Sanger sequencing (Baseclear, Leiden, The Netherlands). The enzymatically inactive pMX-ZFA-MutPRDM9 mutant (G_278_ to A_278_) was obtained by site-directed mutagenesis on wild-type pMX-ZFA-PRDM9. The enzymatically inactive dCas9-MutDOT1L mutant (NN_241–242_ to AD_241–242_) was obtained by site-directed mutagenesis on wild-type dCas9-DOT1L.

### Cell culture

Human embryonic kidney cells HEK293T (ATCC: CRL-3216), A549 lung cancer cells (ATCC: CCL-185), A2780 ovarian cancer cells, and HeLa (ATCC: CCL-2) and C33a (ATCC: HTB-31) cervical cancer cells were cultured in DMEM (BioWhittaker, Walkersville, MD, USA) supplemented with 10% fetal bovine serum (FBS), 2 mM L-glutamine and 50 μg ml^−1^ gentamycine sulfate. Cells were cultured in a humidified atmosphere at 37 °C supplemented with 5% CO_2_. All cell lines have been tested for mycoplasma contamination and authenticated using short tandem repeats (STR) profiling.

### Transfections and retroviral transductions

HEK293T cells were co-transfected with the retroviral vector pMX-IRES-GFP along with VSV-G viral envelope (pMD2.G) and the gag/pol proteins (pMDLg/pRRE) using CaPO_4_. Forty-eight hours and 72 h after transfection, the viral supernatant was used to transduce host cells supplemented with FBS and 5 μg ml^−1^ polybrene (Sigma, St Louis, MO, USA). Cells were collected for further experiments 3 days after the last transduction. Transfections were performed in triplicate using Lipofectamine LTX (Life Technologies). A total of 500,000 cells were seeded into six-well plates the day before transfection. For all experiments, a total of 1 μg of a combination of gRNA plasmids and 1 μg of the plasmid encoding either dCas9-VP64, dCas9-Empty (no effector domain) or (a combination of dCas9-epi-editor(s), were co-transfected using 2 μl PLUS reagent and 4 μl Lipofectamine LTX. GFP positivity of cells was assessed on a Calibur Flow Cytometer (Beckton Dickenson Biosciences).

### Generation of HeLa and C33a stable cell lines

Retroviral particles from pRetroX-Tet-On-Advanced (pTet-On; CloneTech, Mountain View, CA, USA) were generated using conventional CaPO_4_ transfection of HEK293T. Virus-containing supernatant was collected 48 and 72 h post transfection, supplemented with FBS and 5 μg ml^−1^ polybrene, and used to transduce HeLa and C33a cells. Two days after transduction, cells were selected with 1 μg ml^−1^ geneticin (Gibco/Invitrogen) for 5 days and individual clones were subcultured for testing using the pRetroX-Tight-Luc-Pur. The clone with the highest expression of luciferase after induction was chosen for subsequent use. The coding regions of the fusion proteins of ZF_A_, ZF_B_, ZF_2_ and ZF_8_ with VP64, PRDM9 and mutPRDM9 were subcloned into the expression vector pRetroX-Tight-Pur (CloneTech) using the BamHI/NotI restriction sites. Retroviral transduction of the plasmids was carried out as described previously using the stable pTet-On HeLa and C33a cells. Two days after transduction, cells were selected with 1 μg ml^−1^ geneticin (Gibco/Invitrogen) for 10 days. Expression of the fusion proteins was induced using Dox (100 μg ml^−1^) for 72 h. Cells were then collected and divided for RNA, DNA, protein and ChIP, subcultured for 7 days without Dox, and collected again.

### Quantitative reverse-transcription PCR

Total RNA was isolated using the GeneJET RNA Purification kit (Thermo Scientific) according to the protocol. Subsequently, cDNA was synthesized with random hexamer primers using the Revertaid cDNA synthesis kit (Thermo Scientific). qPCR with reverse transcription (qRT–PCR) was executed using 10 ng of cDNA and Rox enzyme mix (Thermo Scientific) for *ICAM1* or *EpCAM* expression (Taqman gene expression assay Hs00164932_m1 or Hs00158980_m1, respectively; Applied Biosystems) and for *GAPDH*, *RASSF1a* and *PLOD2* (Supplementary Table 4) using an ABI ViiA7 real-time PCR system (Applied Biosystems). Expression of the *RASSF1A* and *PLOD2* genes was assessed using ABsolute qPCR SYBR Green (Thermo Scientific). All reactions were done in triplicate. In order to achieve a signal with the qRT–PCR, we run the PCR for 45 cycles. CT values were acquired for all samples, allowing quantitative analysis. Fold change in messenger RNA expression above control untreated cells was calculated based on the cycle threshold (ΔΔCt) method after normalization to *GAPDH* expression.

### ChIP–qPCR

ChIP was performed as described previously^43^. Briefly, fixation of cells was done with formaldehyde and DNA was subsequently sheared by sonification with a Bioruptor (high, 15 cycles: 30′′on, 30′′off) (Diagenode, Liège, Belgium). For immunoprecipitation, Dynabeads (Invitrogen) were incubated 15 min with 5 μg of specific antibodies H3K4me3 (07–473; Merck-Millipore, The Netherlands), H3K79me2 (ab3594; Abcam), H3K79me3 (ab2621; Abcam), normal rabbit IgG (ab46540; Abcam), normal mouse IgG (12–371; Merck-Millipore), anti-FLAG (f1804; Sigma-Aldrich) and anti-HA (101P-200; Covance, The Netherlands)) in 0.02% PBS–Tween-20, then unbound antibodies were washed off, and diluted sheared chromatin was added to the complex of magnetic Dynabeads-antibody (rotating overnight at 4 °C). After washing and elution with 2% SDS and 50 mmol l^−1^ NaHCO_3_, samples were treated with RNase and Proteinase K (Roche). DNA was purified using the Qiaquick DNA spin columns (Qiagen, Venlo, Netherlands) according to the protocol. Subsequently, RT–PCR was performed with AbsoluteQPCR SYBRGreenROXMix (Thermo Scientific) using specific primers (Supplementary Table 5). The % input was expressed as AE (Ct input−Ct ChIP) × Fd × 100%, where Fd is a dilution compensatory factor and AE represents the primer efficiency.

### Western blotting

Cells were lysed in RIPA buffer (25 mM Tris–HCl (pH 7.6), 150 mM NaCl, 1% NP-40, 1% sodium deoxycholate and 0.1% SDS; Thermo Scientific) supplemented with Protease Inhibitor Cocktail (Sigma). An amount of 50 μg of total protein was prepared in 5 × loading buffer supplemented with 10% β-mercaptoethanol and heated for 10 min at 95 °C. Proteins were subjected to SDS–polyacrylamide gel electrophoresisusing 10% polyacrylamide gels. Transfer onto the nitrocellulose membranes was followed by probing with mouse anti-HA antibody (Abcam) at a 1:5,000 dilution. Detection of effector domains was performed with horseradish peroxidase-conjugated anti-mouse secondary antibody at a dilution of 1:5,000, followed by incubation with enhanced chemiluminescence (Amersham).

### Methylation analysis by bisulfite sequencing and pyrosequencing

For DNA methylation analysis of the target regions, genomic DNA was extracted using the Quick-gDNA MiniPrep kit (D3007, Zymo Research via Baseclear) and bisulfite converted using the EZ DNA Methylation-Gold Kit (Zymo Research), following the manufacturer's protocol (alternative 2). Bisulfite converted DNA (20 ng) was amplified by PCR in a 25 μl reaction using the Pyromark PCR kit (Qiagen). Pyrosequencing was performed according to the manufacturer's guidelines with a specific sequencing primer on the Pyromark Q24 MD pyrosequencer (Qiagen). Analysis of the percentage of methylation at each CpG was determined using the Pyromark Q24 Software (Qiagen). Bisulfite-specific primers and the pyrosequencing primer information are presented in Supplementary Table 6.

### Statistical analysis

Statistical tests were performed using the Graphpad Prism 5 software (GraphPad Software). All experiments were performed at least three times, unless stated otherwise. Relevant comparisons were evaluated by unpaired, two-tailed *t*-test. A *P* value of <0.05 was considered statistically significant. All data are presented as the mean±s.d.

### Data availability

The authors declare that all data supporting the findings of this study are available within the article and its Supplementary Information files.

## Additional information

**How to cite this article**: Cano-Rodriguez, D. *et al*. Writing of H3K4Me3 overcomes epigenetic silencing in a sustained but context-dependent manner. *Nat. Commun.* 7:12284 doi: 10.1038/ncomms12284 (2016).

## Supplementary Material

Supplementary Information Supplementary Figures 1-4, Supplementary Tables 1-6 and Supplementary Note 1
