# Supplementary material for: Writing of H3K4Me3 overcomes epigenetic silencing in a sustained but context-dependent manner
Source: Nat Commun. 2016 Aug 10;7:12284. doi: 10.1038/ncomms12284 (PMC4987519; doi:10.1038/ncomms12284)
Supplement: Supplementary Information — Supplementary Figures 1-4, Supplementary Tables 1-6 and Supplementary Note 1 [file ncomms12284-s1.pdf]

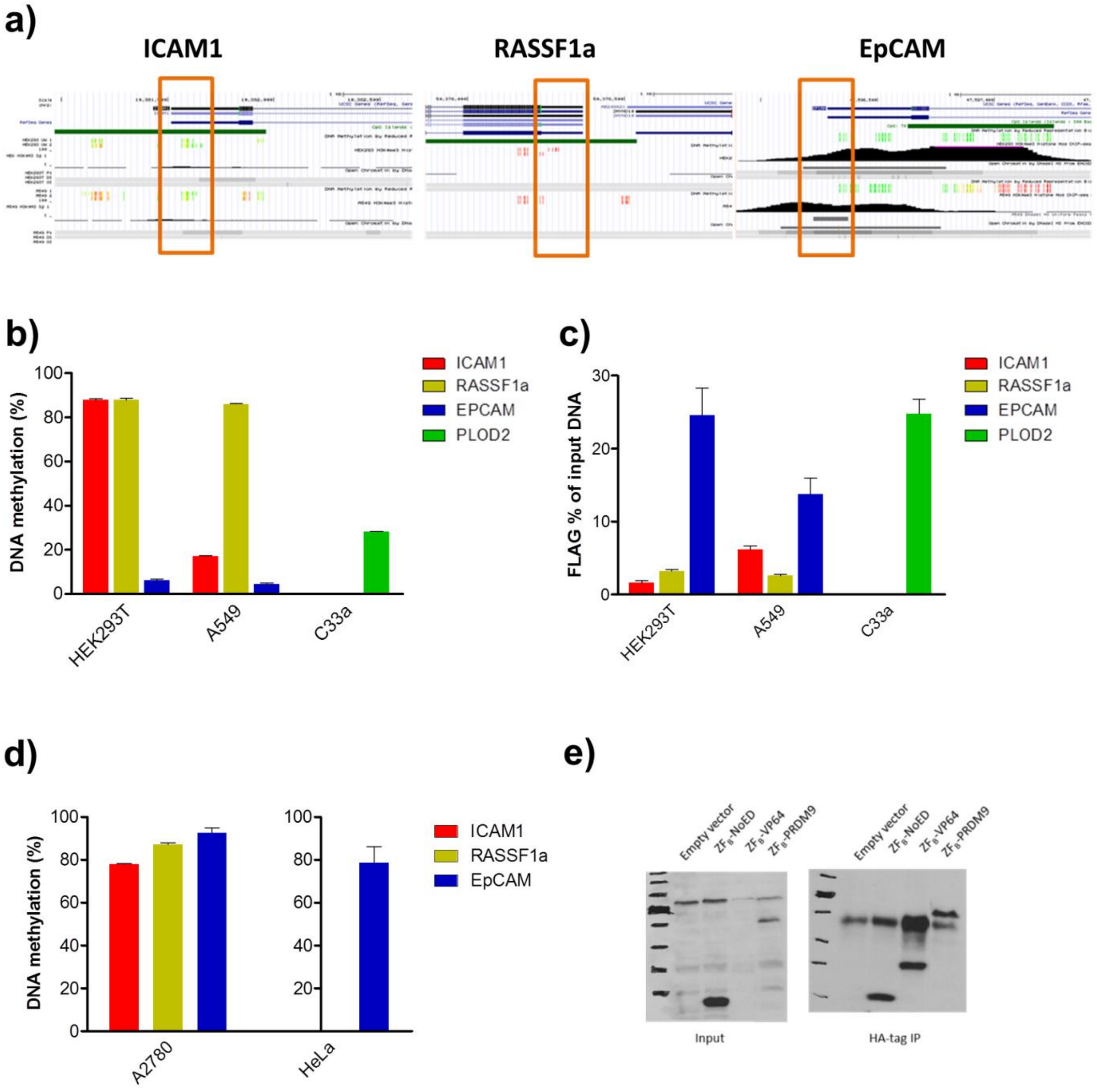

Supplementary Figure 1 Epigenetic landscape of target genes in the studied cell lines (a) ENCODE data depicting the promoter and first exon of the three genes in question. Dark green bars represent the CpG island; light green (unmehtylated), yellow (partially methylated or red (hypermethylated) bars represent DNA methylation profiles; black represent the H3K4me3 peaks of HEK293 and A549; in orange the region targeted with gRNAs (b) DNA methylation levels determined by pyrosequencing of the promoter area. Bars represent the mean methylation of 7-10 CpGs (c) FLAG ChIP-qPCR enrichment at the promoter region of 4 genes compared to levels of DNA methylation in three different cell lines (d) DNA methylation levels determined by pyrosequencing of the promoter area of A2780 (all genes) and HeLA (EpCAM). Bars represent the mean methylation of 7-10 CpGs (e) Protein expression of ZF-fusions in A2780 cells. (*n* = 3 independent experiments; error bars ± s.d.)

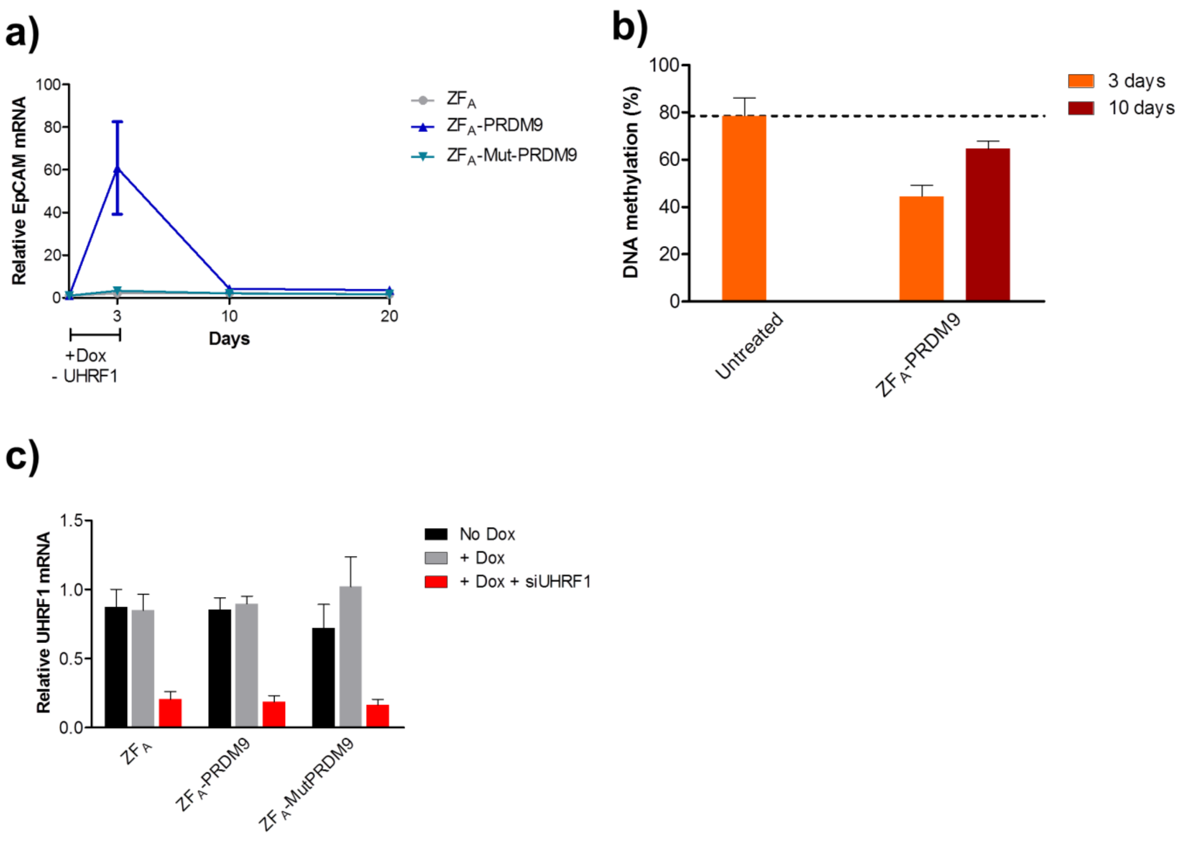

Supplementary Figure 2 Downregulation of key regulator of epigenetic memory through maintenance of DNA methylation after replication (a) Relative *EpCAM* mRNA expression, at each specific time point after Dox and downregulation of UHRF1 for 3 days (b) DNA methylation levels determined by pyrosequencing of the promoter area. Bars represent the mean methylation of 7-10 CpGs (c) Relative *UHRF1* expression after siRNA treatment in all the stable cell lines and treatments. (n = 3 independent experiments; error bars ± s.d.)

a)

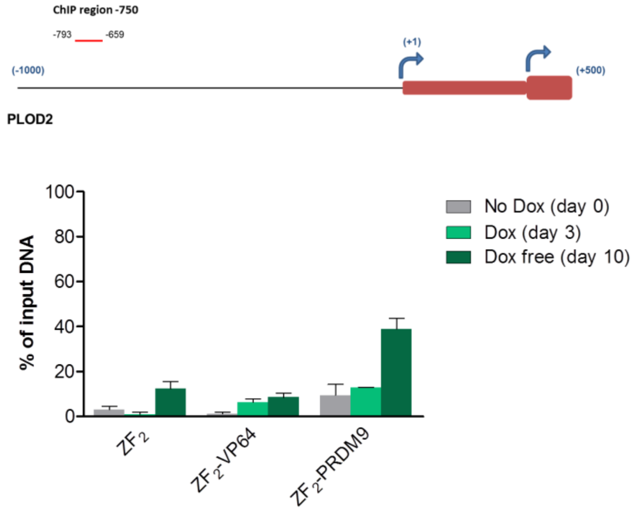

Supplementary Figure 3 Spreading of H3K4me3 750 bps upstream of targeting region (a) H3K4me3 ChIP-qPCR enrichment at the region 750 bps upstream of the PLOD2 promoter. (*n* = 3 independent experiments; error bars ± s.d.)

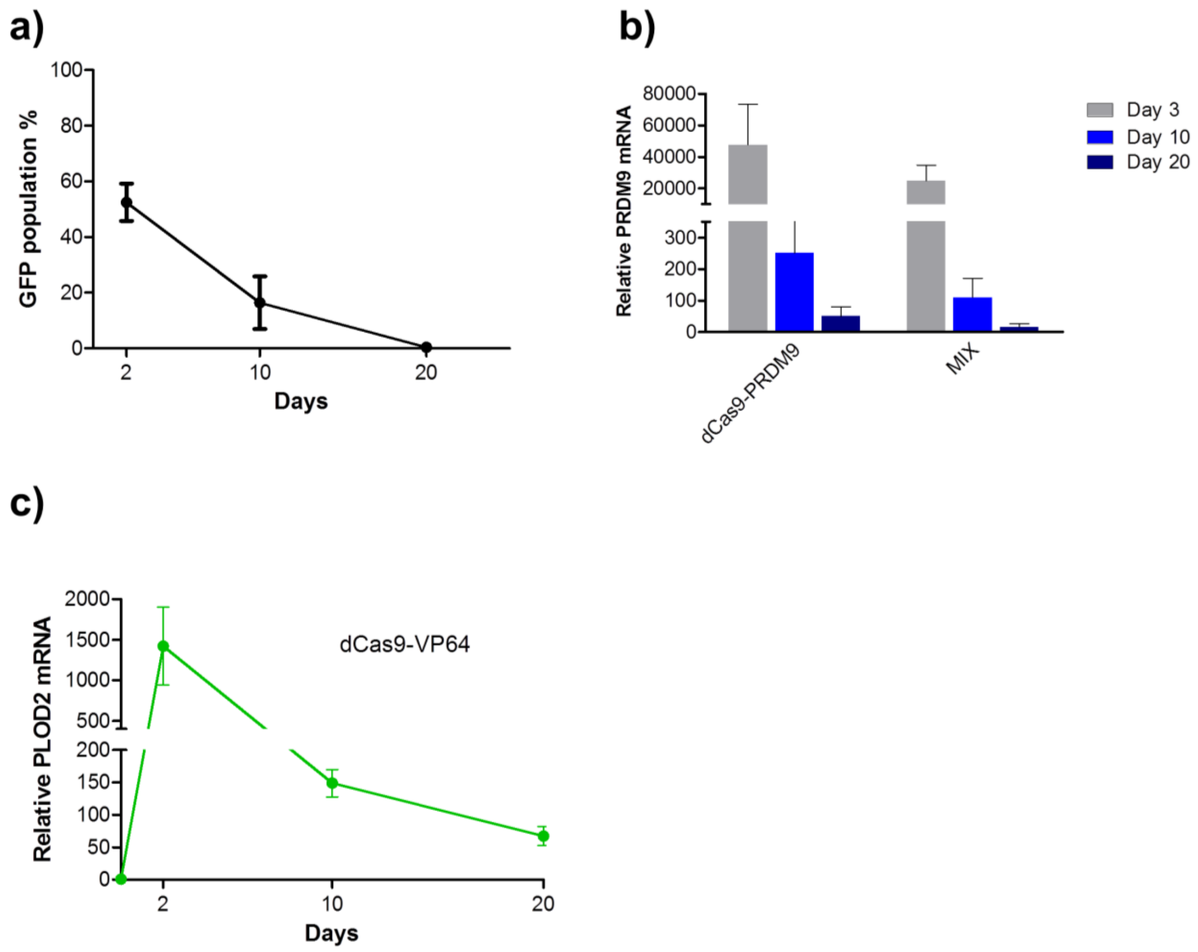

Supplementary Figure 4 (a) Transfection efficiency and transition after subculturing for C33a cells measured as GFP using a CMV-GFP plasmid. (b) Relative expression of PRMD9 in Hela cells treated with 5aza and transfected with the dCas9-effector domains shows the loss of PRDM9 expression (c) Relative PLOD2 mRNA, after co-transfection in C33a cells of dCas9 –VP64 and a combination of gRNAs. (*n* = 3 independent experiments; error bars ± s.d.)

Supplementary Table 1. Primers for effector domain amplification.

Effector domain primers		
**Target**	**Forward Primer (5’-3’)**	**Reverse Primer (5’-3’)**
PRDM9	CTGACGCGTATGGCCTTAAGAGTGGAACA	GGTTAATTAAAGAGGAGTGATTGCGTTCTA
DOT1L	TTAACGCGTGGGGAGAAGCTGGAGCTG	CGTTAATTAATTGGTTCTTCTTGGGCTT
UBE2A	TTAACGCGTATGTCCACCCCGGCTCGG	CGTTAATTAAACAATCACGCCAGCTTTG
MutPRDM9	CTTTGGCCCTTATGAGGCCCGAATTACAGAAGACG	CGTCTTCTGTAATTCGGGCCTCATAAGGGCCAAAAG
MutDOT1L	CACCTCAGGACCAAAGGCAAAATCAGCCACAAATATA	CCAACACGAGTGTTATATTTGTGGCTGATTTTGCCTTTGGTCCTGAGGTG

Supplementary Table 2. gRNA information (in bold the regions shared between gRNA and zinc finger proteins).

***Sp*-dCas9**		
**Target Location**	**Protospacer Sequence (5’- 3’)**	**Genomic Location (GRCh38/hg19 Assembly)**
EPCAM A+	CAGC**GCCGGGGCTGGGGGAG**	Chr2: 47,369,152-47,369,172
EPCAM A-	**CCAGCCCCGGC**GCTGCAGTT	Chr2: 47,369,147-47,369,167
EPCAM B+	CT**ACTCACTCCCCCAACTCC**	Chr2: 47,369,179-47,369,198
EPCAM B-	CG**GGAGTTGGGGGAGTGAGT**	Chr2: 47,369,181-47,369,200
ICAM C+	**CGGAGCTGAAGCGGCC**AGCG	Chr19: 10,270,941-10,270,961
ICAM C-	CTCGCT**GGCCGCTTCAGCTC**	Chr19: 10,270,944-10,270,963
RASSF1a X+	TGTGA**GGAGGGGACGAAGGA**	Chr3: 50,341,033-50,341,052
RASSF1a X-	**TCCT**CACACCCCACCCCGGA	Chr3: 50,341,045-50,341,064
RASSF1a Z+	TGGGGGA**GGCGCTGAAGTCG**	Chr3: 50,340,881-50,340,900
RASSF1a Z-	**CCGACTTCAGCGCC**TCCCCC	Chr3: 50,340,880-50,340,899
RASSF1a Y+	AAGGAAGGGCAAGGCGGGGG	Chr3: 50,341,010-50,341,029
PLOD2 g2	**GCTGTGGAAGCT**ACCGGGGC	Chr3: 146,161,525-146,161,544
PLOD2 g1	CCACTCCCAAAGCTAAGTGC	Chr3: 146,161,426-146,161,445
PLOD2 g3	GAGCCTCCACACGTAGCCGC	Chr3: 146,161,374-146,161,393
PLOD2 g3	TGAGCAAACAGTCCAGACGT	Chr3: 146,161,327-146,161,346

Supplementary Table 3. Zinc finger information.

**ZFP**		
**Target Location**	**Target site (5’- 3’)**	**Genomic Location (GRCh38/hg19 Assembly)**
EPCAM A	**GCCGGGGCTGGGGGAG**GG	Chr2: 47,369,157-47,369,174
EPCAM B	**ACTCACTCCCCCAACTCC**	Chr2: 47,369,181-47,369,198
ICAM CD54	TC**CGGAGCTGAAGCGGCC**	Chr19: 10,270,940-10,270,957
RASSF1a X	**GGAGGGGACGAAGGA**GGG	Chr3: 50,341,030-50,341,047
RASSF1a Z	**GGCGCTGAAGTCG**GGGCC	Chr3: 50,340,876-50,340,893
PLOD2 ZF2	GAGGCT**GCTGTGGAAGCT**	Chr3: 146,161,533-146,161,550
PLOD2 ZF8	GGGGCCGTGGGCGCCTGA	Chr3: 146,161,016-146,161,033

Supplementary Table 4. Quantitative reverse transcription PCR

qRT-PCR primers			
**Target**	**Forward Primer (5’-3’)**	**Reverse Primer (5’-3’)**	**Probe**
GAPDH	CCACATCGCTCAGACACCAT	GCGCCCAATACGACCAAAT	CGTTGACTCCGACCTTCACCTTCCC
RASSF1a	AAGTTCACCTGCCACTACCG	AAGGTCAGGTGTCTCCCACT	
PLOD2	GGGAGTTCATTGCACCAGTT	GAGGACGAAGAGAACGC	

Supplementary Table 5. ChIP-qPCR primers

	ChIP primers		
**Target**	**Forward Primer (5'-3')**	**Reverse Primer (5'-3')**	**Probe**
EPCAM ChIP	CCCAACTCCCGGGCGGTGACT	TGTCCTCCCGACGCGGACCC	
RASSF1a ChIP	TGGGGTGTGAGGAGGGGACGA	AGAGCCGCGCAATGGA	
PLOD2 ChIP region 2	GAGGCTGCTGTGGAAGCTA	GGTTTGGGAGAGGGAGGAG	AGACGGGAACACCGCCCTCC
PLOD2 ChIP region 8	CACCGACGACCTCACTCA	TCGCGAGAACGCAGAGAC	CTGCGTTACGCGCCGCTC
PLOD2 ChIP region -750	ACAAAACGTGATCATAATGGAA	ATTTAGGCAGGAGATTCCAGAA	

Supplementary Table 6. Pyrosequencing primers

	Pyrosequencing primers		
**Target**	**Forward Primer (5'-3')**	**Reverse Primer (5'-3')**	**Pyrosequencing**
ICAM1	GGGGAAGTTGGTAGTATTTAAAAGT	[Btn] CCTTCCCCTCCCAAACAAATACTACAATTA	GTTAGATTGTTTTAGT
EPCAM	TGGGGGAGGGGAGTTTATT	[Btn] ACCCAACTCCACAACTCT	AGGGGAGTTTATTTATTTTTTTA
RASSF1a	AAGGAGGGAAGGAAGGGTAAG	gggacaccgctgatcgtttaCCCCCAACTCAATAAACTCAAACTCCCC	GAAGGAAGGGTAAGG
PLOD2	AAGTAGTGGGGGTTAAGG	[Btn] CCAAAAAAACCTTAAACTCTTTCCAATAAT	GGGTTAAGGTTTAGAGA
Universal	[Btn] gggacaccgctgatcgttta		

Supplementary Note 1. CCDS Sequences of amplified epigenetic enzymes (in-between green is amplified region, in red the nucleotides changed for site directed mutagenesis)

PRDM9

ATGAGCCCTGAAAAGTCCCAAGAGGAGAGCCCAGAAGAAGACACAGAGAGAACAGAGCGGAAGCCCATGGT
CAAAGATGCCTTCAAAGACATTTCCATATACTTCACCAAGGAAGAATGGGCAGAGATGGGAGACTGGGAGAA
AACTCGCTATAGGAATGTGAAAAGGAACTATAATGCACTGATTACTATAGGTCTCAGAGCCACTCGACCAGCT
TTCATGTGTCACCGAAGGCAGGCCATCAAACTCCAGGTGGATGACACAGAAGATTCTGATGAAGAATGGACC
CCTAGGCAGCAAGTCAAACCTCCTTGGATGGCCTTAAGAGTGGAACAGCGTAAACACCAGAAGGGAATGCC
CAAGGCGTCATTCAGTAATGAATCTAGTTTGAAAGAATTGTCAAGAACAGCAAATTTACTGAATGCAAGTGGC
TCAGAGCAGGCTCAGAAACCAGTGTCCCCTTCTGGAGAAGCAAGTACCTCTGGACAGCACTCAAGACTAAAA
CTGGAACTCAGGAAGAAGGAGACTGAAAGAAAGATGTATAGCCTGCGAGAAAGAAAGGGTCATGCATACAAA
GAGGTCAGCGAGCCGCAGGATGATGATTACCTCTATTGTGAGATGTGTCAGAACTTCTTCATTGACAGCTGT
GCTGCCCATGGGCCCCCTACATTTGTAAAGGACAGTGCAGTGGACAAGGGGCACCCCAACCGTTCAGCCCT
CAGTCTGCCCCCAGGGCTGAGAATTGGGCCATCAGGCATCCCTCAGGCTGGGCTTGGAGTATGGAATGAGG
CATCTGATCTGCCGCTGGGTCTGCACTTTGGCCCTTATGAGGGCCGAATTACAGAAGACGAAGAGGCAGCC
AACAATGGATACTCCTGGCTGATCACCAAGGGGAGAAACTGCTATGAGTATGTGGATGGAAAAGATAAATCC
TGGGCCAACTGGATGAGGTATGTGAACTGTGCCCGGGATGATGAAGAGCAGAACCTGGTGGCCTTCCAGTA
CCACAGGCAGATCTTCTATAGAACCTGCCGAGTCATTAGGCCAGGCTGTGAACTGCTGGTCTGGTATGGGG
ATGAATACGGCCAGGAACTGGGCATCAAGTGGGGCAGCAAGTGGAAGAAAGAGCTCATGGCAGGGAGAGA
ACCAAAGCCAGAGATCCATCCATGTCCCTCATGCTGTCTGGCCTTTTCAAGTCAGAAATTTCTCAGTCAACAT
GTAGAACGCAATCACTCCTCTCAGAACTTCCCAGGACCATCTGCAAGAAAACTCCTCCAACCAGAGAATCCC
TGCCCAGGGGATCAGAATCAGGAGCAGCAATATCCAGATCCACACAGCCGTAATGACAAAACCAAAGGTCAA
GAGATCAAAGAAAGGTCCAAACTCTTGAATAAAAGGACATGGCAGAGGGAGATTTCAAGGGCCTTTTCTAGC
CCACCCAAAGGACAAATGGGGAGCTGTAGAGTGGGAAAAAGAATAATGGAAGAAGAGTCCAGAACAGGCCA
GAAAGTGAATCCAGGGAACACAGGCAAATTATTTGTGGGGGTAGGAATCTCAAGAATTGCAAAAGTCAAGTA
TGGAGAGTGTGGACAAGGTTTCAGTGTTAAATCAGATGTTATTACACACCAAAGGACACATACAGGGGAGAA
GCTCTACGTCTGCAGGGAGTGTGGGCGGGGCTTTAGCTGGAAGTCACACCTCCTCATTCACCAGAGGATAC
ACACAGGGGAGAAGCCCTATGTCTGCAGGGAGTGTGGGCGGGGCTTTAGCTGGCAGTCAGTCCTCCTCACT
CACCAGAGGACACACACAGGGGAGAAGCCCTATGTCTGCAGGGAGTGTGGGCGGGGCTTTAGCCGGCAGT
CAGTCCTCCTCACTCACCAGAGGAGACACACAGGGGAGAAGCCCTATGTCTGCAGGGAGTGTGGGCGGGG
CTTTAGCCGGCAGTCAGTCCTCCTCACTCACCAGAGGAGACACACAGGGGAGAAGCCCTATGTCTGCAGGG
AGTGTGGGCGGGGCTTTAGCTGGCAGTCAGTCCTCCTCACTCACCAGAGGACACACACAGGGGAGAAGCC
CTATGTCTGCAGGGAGTGTGGGCGGGGCTTTAGCTGGCAGTCAGTCCTCCTCACTCACCAGAGGACACACA
CAGGGGAGAAGCCCTATGTCTGCAGGGAGTGTGGGCGGGGCTTTAGCAATAAGTCACACCTCCTCAGACAC
CAGAGGACACACACAGGGGAGAAGCCCTATGTCTGCAGGGAGTGTGGGCGGGGCTTTCGCGATAAGTCAC
ACCTCCTCAGACACCAGAGGACACACACAGGGGAGAAGCCCTATGTCTGCAGGGAGTGTGGGCGGGGCTT
TAGAGATAAGTCAAACCTCCTCAGTCACCAGAGGACACACACAGGGGAGAAGCCCTATGTCTGCAGGGAGT
GTGGGCGGGGCTTTAGCAATAAGTCACACCTCCTCAGACACCAGAGGACACACACAGGGGAGAAGCCCTAT
GTCTGCAGGGAGTGTGGGCGGGGCTTTCGCAATAAGTCACACCTCCTCAGACACCAGAGGACACACACAGG
GGAGAAGCCCTACGTCTGCAGGGAGTGTGGGCGGGGCTTTAGCGATAGGTCAAGCCTCTGCTATCACCAGA
GGACACACACAGGGGAGAAGCCCTACGTCTGCAGGGAGGATGAGTAA

DOT1L

ATGGGGGAGAAGCTGGAGCTGAGACTGAAGTCGCCCGTGGGGGCTGAGCCCGCCGTCTACCCGTGGCCG
CTGCCGGTCTACGATAAACATCACGATGCTGCTCATGAAATCATCGAGACCATCCGATGGGTCTGTGAAGAA
ATCCCGGATCTCAAGCTCGCTATGGAGAATTACGTTTTAATTGACTATGACACCAAAAGCTTCGAGAGCATGC
AGAGGCTCTGCGACAAGTACAACCGTGCCATCGACAGCATCCACCAGCTGTGGAAGGGCACCACGCAGCCC
ATGAAGCTGAACACGCGGCCGTCCACTGGACTCCTGCGCCATATCCTGCAGCAGGTCTACAACCACTCGGT
GACCGACCCCGAGAAGCTCAACAACTACGAGCCCTTCTCCCCCGAGGTGTACGGGGAGACCTCCTTCGACC
TGGTGGCCCAGATGATTGATGAGATCAAGATGACCGACGACGACCTGTTTGTGGACTTGGGGAGCGGTGTG
GGCCAGGTCGTGCTCCAGGTTGCTGCTGCCACCAACTGCAAACATCACTATGGCGTCGAGAAAGCAGACAT
CCCGGCCAAGTATGCGGAGACCATGGACCGCGAGTTCAGGAAGTGGATGAAATGGTATGGAAAAAAGCATG
CAGAATACACATTGGAGAGAGGCGATTTCCTCTCAGAAGAGTGGAGGGAGCGAATCGCCAACACGAGTGTT
ATATTTGTGAATAATTTTGCCTTTGGTCCTGAGGTGGATCACCAGCTGAAGGAGCGGTTTGCAAACATGAAGG

AAGGTGGCAGAATCGTGTCCTCGAAACCCTTTGCACCTCTGAACTTCAGAATAAACAGTAGAAACTTGAGTGA
CATCGGCACCATCATGCGCGTGGTGGAGCTCTCGCCCCTGAAGGGCTCGGTGTCGTGGACGGGGAAGCCA
GTCTCCTACTACCTGCACACTATCGACCGCACCATACTTGAAAACTATTTTTCTAGTCTGAAAAACCCAAAACT
CAGGGAGGAACAGGAGGCAGCCCGGCGCCGCCAGCAGCGCGAGAGCAAGAGCAACGCGGCCACGCCCAC
TAAGGGCCCAGAGGGCAAGGTGGCCGGCCCCGCCGACGCCCCCATGGACTCTGGTGCTGAGGAAGAGAA
GGCGGGAGCAGCCACCGTGAAGAAGCCGTCTCCCTCCAAAGCCCGCAAGAAGAAGCTAAACAAGAAGGGG
AGGAAGATGGCTGGCCGCAAGCGCGGGCGCCCCAAGAAGATGAACACTGCGAACCCCGAGCGGAAGCCCA
AGAAGAACCAAACTGCACTGGATGCCCTGCACGCTCAGACCGTGTCTCAGACGGCGGCCTCCTCACCCCAG
GATGCCTACAGATCCCCTCACAGCCCGTTCTACCAGCTACCTCCGAGCGTGCAGCGGCACTCCCCCAACCC
GCTGCTGGTGGCGCCCACCCCGCCCGCGCTGCAGAAGCTTCTAGAGTCCTTCAAGATCCAGTACCTGCAGT
TCCTGGCATACACAAAGACCCCCCAGTACAAGGCCAGCCTGCAGGAGCTGCTGGGCCAGGAGAAGGAGAA
GAACGCCCAGCTCCTGGGTGCGGCTCAGCAGCTCCTCAGCCACTGCCAGGCCCAGAAGGAGGAGATCAGG
AGGCTGTTTCAGCAAAAATTGGATGAGCTGGGTGTGAAGGCGCTGACCTACAACGACCTGATTCAAGCGCA
GAAGGAGATCTCCGCCCATAACCAGCAGCTGCGGGAGCAGTCGGAGCAGCTGGAGCAGGACAACCGCGCG
CTCCGCGGCCAGAGCTTGCAGCTGCTCAAGGCTCGCTGCGAGGAGCTGCAGCTGGACTGGGCCACGCTGT
CGCTGGAGAAGCTGTTGAAGGAGAAGCAGGCCCTGAAGAGCCAGATCTCGGAGAAGCAGAGGCACTGCCT
GGAGCTGCAGATCAGCATTGTGGAGCTAGAGAAGAGCCAGCGGCAGCAGGAGCTCCTGCAGCTCAAGTCCT
GTGTGCCGCCTGACGACGCCCTGTCCCTGCACCTGCGTGGGAAGGGCGCCCTGGGCCGCGAGCTGGAGC
CTGACGCCAGCCGGCTGCACCTGGAGCTGGACTGCACCAAGTTCTCGCTGCCTCACTTGAGCAGCATGAGC
CCGGAGCTCTCCATGAACGGCCAGGCTGCTGGCTATGAGCTCTGCGGTGTGCTGAGCCGGCCTTCGTCGAA
GCAGAACACGCCCCAGTACCTGGCCTCACCCCTGGACCAGGAGGTGGTGCCCTGTACCCCTAGCCACGTC
GGCCGGCCGCGCCTGGAGAAGCTGTCTGGCCTAGCCGCACCCGACTACACTAGGCTGTCCCCGGCCAAGA
TTGTGCTGAGGCGGCACCTGAGCCAGGACCACACGGTGCCCGGCAGGCCGGCTGCCAGTGAGCTGCATTC
GAGAGCTGAGCACACCAAGGAGAACGGCCTTCCCTACCAGAGCCCCAGCGTGCCTGGCAGCATGAAGCTG
AGCCCTCAGGACCCGCGGCCCCTGTCCCCTGGGGCCTTGCAGCTTGCTGGAGAGAAGAGCAGTGAGAAGG
GCCTGAGAGAGCGCGCCTACGGCAGCAGCGGGGAGCTCATCACCAGCCTGCCCATCAGCATCCCGCTCAG
CACCGTGCAGCCCAACAAGCTCCCGGTCAGCATTCCCCTGGCCAGCGTGGTGCTGCCCAGCCGCGCCGAG
AGGGCGAGGAGCACCCCCAGTCCCGTGCTGCAGCCCCGTGACCCCTCGTCCACACTTGAAAAGCAGATTG
GTGCTAATGCCCACGGTGCTGGGAGCAGAAGCCTTGCCCTGGCCCCCGCAGGCTTCTCCTACGCTGGCTC
GGTGGCCATCAGCGGGGCCTTGGCGGGCAGCCCGGCCTCTCTCACACCTGGAGCCGAGCCGGCCACCTTG
GATGAGTCCTCCAGCTCTGGGAGCCTTTTTGCCACCGTGGGGTCCCGCAGCTCCACGCCACAGCACCCCCT
GCTGCTGGCACAGCCCCGGAACTCGCTTCCTGCCTCTCCCGCCCACCAGCTCTCCTCCAGTCCCCGGCTTG
GTGGGGCCGCCCAGGGCCCGTTGCCCGAGGCCAGCAAGGGAGACCTGCCCTCCGATTCCGGCTTCTCAGA
TCCTGAGAGTGAAGCCAAGAGGAGGATTGTGTTCACCATCACCACTGGTGCGGGCAGTGCCAAGCAGTCGC
CCTCCAGCAAGCACAGCCCCCTGACCGCCAGCGCCCGTGGGGACTGTGTGCCGAGCCACGGGCAGGACA
GTCGCAGGCGCGGCCGGCGGAAGCGAGCATCTGCGGGGACGCCCAGCTTGAGCGCAGGCGTGTCCCCCA
AGCGCCGAGCCCTGCCGTCCGTCGCTGGCCTTTTCACACAGCCTTCGGGGTCTCCCCTCAACCTCAACTCC
ATGGTCAGTAACATCAACCAGCCCCTGGAGATTACAGCCATCTCGTCCCCGGAGACCTCCCTGAAGAGCTCC
CCTGTGCCCTACCAGGACCACGACCAGCCCCCCGTGCTCAAGAAGGAGCGGCCTCTGAGCCAGACCAATG
GGGCACACTACTCCCCACTCACCTCAGACGAGGAGCCAGGCTCTGAGGACGAGCCCAGCAGTGCTCGAATT
GAGAGAAAAATTGCAACAATCTCCTTAGAAAGCAAATCTCCCCCGAAAACCTTGGAAAATGGTGGTGGCTTG
GCGGGAAGGAAGCCCGCGCCCGCCGGCGAGCCAGTCAATAGCAGCAAGTGGAAGTCCACCTTCTCGCCCA
TCTCCGACATCGGCCTGGCCAAGTCGGCGGACAGCCCGCTGCAGGCCAGCTCCGCCCTCAGCCAGAACTC
CCTGTTCACGTTCCGGCCCGCCCTGGAGGAGCCCTCTGCCGATGCCAAGCTGGCCGCTCACCCCAGGAAA
GGCTTTCCCGGCTCCCTGTCGGGGGCTGACGGACTCAGCCCGGGCACCAACCCTGCCAACGGCTGCACCT
TCGGCGGGGGCCTGGCCGCGGACCTGAGTTTACACAGCTTCAGTGATGGTGCTTCTCTTCCCCACAAGGGC
CCCGAGGCGGCCGGCCTGAGCTCCCCGCTGAGCTTCCCCTCGCAGCGCGGCAAGGAGGGCTCGGACGCC
AACCCTTTCCTGAGCAAGAGGCAGCTGGACGGCCTGGCTGGGCTGAAGGGCGAGGGCAGCCGCGGCAAG
GAGGCAGGGGAGGGCGGCCTACCGCTGTGCGGGCCCACGGACAAGACCCCACTGCTGAGCGGCAAGGCC
GCCAAGGCCCGGGACCGCGAGGTCGACCTCAAGAATGGCCACAACCTCTTCATCTCTGCGGCGGCCGTGC
CTCCCGGAAGCCTCCTCAGCGGCCCCGGCCTGGCCCCGGCGGCGTCCTCCGCAGGCGGCGCGGCGTCCT
CCGCCCAGACGCACCGGTCCTTCCTGGGCCCCTTCCCGCCGGGACCGCAGTTCGCGCTCGGCCCCATGTC
CCTGCAGGCCAACCTCGGCTCCGTGGCCGGCTCCTCCGTGCTGCAGTCGCTGTTCAGCTCTGTGCCGGCC
GCCGCAGGCCTGGTGCACGTGTCGTCCGCTGCCACCAGACTGACCAACTCGCACGCCATGGGCAGCTTTTC
CGGGGTGGCAGGCGGCACAGTTGGAGGTAACTAG

UBE2A

ATGTCCACCCCGGCTCGGCGGCGCCTCATGCGGGACTTCAAGAGGTTGCAGGAGGATCCTCCAGCCGGAG
TCAGCGGGGCTCCGTCCGAGAACAACATAATGGTGTGGAACGCGGTCATTTTCGGGCCTGAAGGGACCCCG
TTTGAGGATGGAACATTTAAACTTACAATAGAATTCACTGAAGAATATCCAAATAAACCACCTACAGTTAGATT
TGTCTCTAAGATGTTCCATCCAAATGTCTATGCAGATGGTAGTATATGTCTGGACATACTTCAGAACCGTTGG
AGTCCAACCTATGATGTGTCTTCCATTCTAACATCCATACAGTCTCTGTTGGATGAACCCAATCCCAATAGTC
CAGCAAACAGCCAGGCTGCTCAGCTGTACCAGGAGAACAAACGGGAATATGAAAAGCGTGTTTCTGCAATAG
TAGAACAAAGCTGGCGTGATTGT
